# Detection of antibodies against H5 and H7 strains in birds: evaluation of influenza pseudovirus particle neutralization tests

**DOI:** 10.3402/iee.v4.23011

**Published:** 2014-01-15

**Authors:** Sofie Wallerström, Nina Lagerqvist, Nigel J. Temperton, Michaela Cassmer, Ana Moreno, Malin Karlsson, Mikael Leijon, Åke Lundkvist, Kerstin I. Falk

**Affiliations:** 1 Karolinska Institutet, Department of Microbiology, Tumor and Cell Biology, Stockholm, Sweden; 2 Swedish Institute for Communicable Disease Control, Department of Diagnostics and Vaccinology, Solna, Sweden; 3 Viral Pseudotype Unit, University of Kent, Kent, UK; 4 Reparto di Virologia, Istituto Zooprofilattico Sperimentale della Lombardia ed Emilia Romagna, Brescia, Italy; 5 National Veterinary Institute, Swedish University of Agricultural Sciences, Uppsala, Sweden; 6 Department of Medical Biochemistry and Microbiology, Uppsala University, Uppsala, Sweden

**Keywords:** influenza A, pseudovirus, neutralization, antibodies, avian

## Abstract

**Introduction:**

Avian influenza viruses circulate in bird populations, and it is important to maintain and uphold our knowledge of the viral strains that are currently of interest in this context. Here, we describe the use of hemagglutinin-pseudotype retroviruses based on highly pathogenic influenza viruses for the screening of avian sera for influenza A antibodies. Our aim was also to determine whether the pseudovirus neutralization tests that we assessed were sensitive and simple to use compared to the traditional methods, including hemagglutination inhibition assays and microneutralization tests.

**Material and methods:**

H5 and H7 pseudovirus neutralization tests were evaluated by using serum from infected rabbits. Subsequently, the assays were further investigated using a panel of serum samples from avian species. The panel contained samples that were seropositive for five different hemagglutinin subtypes as well as influenza A seronegative samples.

**Results and discussion:**

The results suggest that the pseudovirus neutralization test is an alternative to hemagglutination inhibition assays, as we observed comparable titers to those of both standard microneutralizations assays as well as hemagglutinin inhibition assays. When evaluated by a panel of avian sera, the method also showed its capability to recognize antibodies directed toward low-pathogenic H5 and H7. Hence, we conclude that it is possible to use pseudoviruses based on highly pathogenic avian influenza viruses to screen avian sera for antibodies directed against influenza A subtypes H5 and H7.

Influenza A virus (IAV) belongs to the family *Orthomyxoviridae*, and its genome consists of eight RNA segments of negative polarity that together code for a minimum of 10 proteins. Classification of IAV is based on the two surface proteins hemagglutinin (HA) and neuraminidase (NA). Studies have previously described 16 serologically distinct and well-characterized types of HA and nine different types of NA in birds (1–5), and nearly all of the 144 combinations of these two proteins were found in wild dabbling ducks (6, 7). However, in 2009, a novel IAV H17N10 variant was also discovered in fruit bats (8, 9). IAV is endemic in waterfowl, especially in species belonging to the order Anseriformes, and it is particularly prevalent in dabbling ducks of the genus *Anas*, suggesting that this taxon constitutes the natural reservoir (6, 10–12).

Highly pathogenic avian influenza (HPAI) can circulate in wild birds, and it can have a deadly outcome if introduced into domestic poultry (13, 14). Two subtypes of HPAI virus, designated H5 and H7, have been found in birds (15–17). Furthermore, over the past 10 years, 630 cases of H5N1 HPAI have been confirmed in humans, 375 of which led to death (18). Recently, avian influenza of subtype H7N9 has emerged in China, with 132 cases confirmed, 37 of them resulting in death (19). As of yet, the role of migratory waterfowl as vectors of HPAI virus has not been proven or disproven (20–22). Although outbreaks of HPAI virus are extremely rare in wild birds, it is possible that HPAI virus can be maintained in populations of these avian species (23), indicating the importance of surveillance.

Low pathogenic avian influenza (LPAI) virus circulates in wild ducks and is normally not associated with severe disease. Mallards exhibit immune responses when experimentally infected with LPAI virus (24), whereas it has been suggested that infections with LPAI virus in wild birds have only minor clinically measurable effects (25–27). In birds, influenza virus is excreted continuously in feces for up to 12 days (24, 28), and antibodies to IAV can be detected long after viral shedding has ceased (7. Consequently, RT-PCR detection of the shedding of viral RNA is often performed to monitor the current influenza status in birds. A method that is used to detect influenza A antibodies is the hemagglutination inhibition (HI) assay, and it has long been the preferred method for that purpose even though it is well known that HI titers can vary between laboratories (29, 30). As an alternative, HA subtype-specific enzyme-linked immunosorbent assays (ELISAs) can be used to screen sera for IAV antibodies. However, there are no commercially available ELISA kits specific for all 17 of the known HA subtypes, and, as with the HI assay, ELISA cannot specifically detect neutralizing antibodies. Virus neutralization tests, which are usually performed in microformat (microneutralization, or MN) (31), represent another option, but these methods require the use of viable wild-type virus and also biosafety level 3 containment facilities when handling HPAI viruses. Moreover, evaluation of neutralization tests by cytopathic effect (CPE) is time consuming and laborious, and titers can vary between laboratories.

MN tests based on pseudoviruses expressing IAV HA H5 and H7 on the surface have been recognized as reliable and safe alternatives for detection of IAV-neutralizing antibodies (32–39). Another aspect of using pseudovirus particles is the fact that it makes collaborations easier, as sharing plasmids or even viral antigen sequences is preferable over sending live infectious virus. To further examine the prospects of pseudovirus particle neutralization tests (pp-NTs), we performed and evaluated seven pp-NTs using different subtypes of HPAI pseudoviruses (six H5 and one H7) to screen for antibodies against matching subtypes of LPAI virus. The HPAI variants are useful because of the elongated polybasic cleavage site in the HA, which is efficiently cleaved into HA1 and HA2 by cellular proteases (40, 41). Serum from infected rabbits was used to enable comparison of the pp-NTs with the traditional HI assay and with MN tests using CPE as readout, as well as two additional MN tests based on enzyme-catalyzed color development. Further, the pp-NTs were evaluated using a panel of sera obtained from experimentally and naturally infected avian species. The results of this study confirm that pseudoviruses based on HPAI are neutralized by immune serum from both LPAI-H5- and LPAI-H7-infected animals, and that the pp-NTs are a good alternative to the traditional methods used to screen for IAV antibodies in avian sera.

## Materials and methods

### Viruses and retroviral particles pseudotyped with HPAI HA

The protocol for inoculation of hens’ eggs was approved by the Committee on the Ethics of Animal Experiments, Karolinska Institutet, Stockholm, Sweden (permit number N341/06). LPAI virus of the strain A/mallard/Sweden/6566/2004 (H5N2) or A/mallard/Sweden/7206/2004 (H7N7) (GenBank: JN674638) was inoculated into the allantoic cavity of 10-day-old embryonated specific-pathogen-free hens’ eggs. The eggs were then incubated for 72 h at 37°C followed by 12–24 h at 4°C, after which the allantoic fluid was harvested, centrifuged, and stored at −70°C until used. The HA genes of the H5N2 and H7N7 isolates were sequenced by Sanger sequencing according to a previously established protocol (42). The HA pseudotypes were based on the six H5N1 HPAI strains A/Anhui/1/2005, A/HongKong/213/03, A/Indonesia/5/2005, A/whooper swan/Mongolia/244/2005, A/turkey/Turkey/1/2005, and A/Vietnam/1194/2004, as well as the HPAI H7NI strain A/chicken/Italy/13474/99; here, these pseudotypes are simply denoted as Anhui, Hong Kong, Indonesia, Mongolia, Turkey, Vietnam, and Italy (H7), respectively. Reports in the literature give detailed descriptions of the cloning of the full-length HA open reading frame into the expression vector pI.18, the production of retroviral (MLV) vector particles pseudotyped with IVA HA (39), and the MLV gag/pol and firefly luciferase (Luc) reporter MLV–Luc constructs (43, 44).

### Cell lines

Madin–Darby canine kidney (MDCK) cells (ATCC: CCL-34) were cultured in Medium 199 supplemented with 10% fetal bovine serum (FBS), 100 U/mL penicillin, and 100 µg/mL streptomycin. Human kidney–derived 293T/17 cells (ATCC: CRL-11268) were cultured in Dulbecco's Modified Eagle Medium (DMEM) supplemented with 15% FBS and antibiotics (100 U/mL penicillin and 100 µg/mL streptomycin). The cells were maintained at 37°C in a humidified atmosphere with 5% CO_2_. All reagents were purchased from Life Technologies.

### Serum samples

Rabbits were immunized with IAV according to a slightly modified protocol (45). Intranasal infections of rabbits were carried out in strict accordance with the provisions and general guidelines of the Swedish Animal Welfare Agency. The protocol was approved by the Committee on the Ethics of Animal Experiments at Karolinska Institutet, Stockholm (permit number N386/07), and all efforts were made to minimize suffering. Two New Zealand white rabbits were intranasally infected with 1 mL of egg-grown H5N2 or H7N7 virus per nostril, followed by a booster dose 3 weeks later. Before infection, serum samples were collected via an ear vein while the rabbits were under mild anesthesia (Hypnorm), and the animals were sampled and euthanized (Hypnorm/Dormicum) 9 weeks after infection. To confirm subtype specificity of the acquired sera, IAV antibody ELISA (IDEXX Laboratories Inc.) and H5 and H7 subtype-specific ELISAs (ID.vet Innovative Diagnostics) were performed according to the manufacturers’ protocols.

The serum panel consisted of six IAV-negative and 14 IAV-positive samples from five avian species (chicken, duck, partridge, pheasant, and turkey) that had previously been confirmed for subtype specificity by HI assay (Table 1) (46–48).

**Table 1:** Geometric mean titer of influenza antibodies in avian sera determined by pseudovirus particle neutralization testing

Serum				pp-NT(H5)^b^						pp-NT(H7)^b^
			
Strain	Species	Description	HI^a^	Anhui	Hong Kong	Indonesia	Mongolia	Turkey	Vietnam	Italy
H4N6	SPF chicken	Exp. infection	2048	–	–	–	–	–	–	–
H5N1	Turkey	Exp. infection	32	170	200	110	100	80	80	–
H5N1	Turkey	Exp. infection	64	40	80	80	60	290	100	–
H5N2	Pheasant	Field sera	64	90	150	70	200	40	90	–
H5N2	Pheasant	Field sera	64	120	350	60	90	230	150	–
H5N2	Turkey	Field sera	64	2060	90	–	70	50	60	–
H5N9	SPF chicken	Exp. infection	128	150	110	60	50	40	130	–
H6N2	SPF chicken	Exp. infection	32	–	–	–	–	–	–	–
H7N1	Duck	Exp. infection	2048	–	–	–	–	–	–	100
H7N1	Duck	Exp. infection	1024	–	–	–	–	–	–	130
H7N3	Turkey	Field sera	16	–	–	–	–	–	–	–
H7N3	Turkey	Field sera	16	–	–	–	–	–	–	–
H7N7	SPF chicken	Exp. infection	256	–	–	–	–	–	–	40
H9N2	Duck	Exp. infection	32	–	–	–	–	–	–	–
Neg	Turkey	Field sera	<2	–	–	–	–	–	–	–
Neg	Turkey	Field sera	<2	–	–	–	–	–	–	–
Neg	Turkey	Field sera	<2	–	–	–	–	–	–	–
Neg	Partridge	Field sera	<2	–	–	–	–	–	–	–
Neg	SPF chicken	Field sera	<2	–	–	–	–	–	–	–
Neg	Chicken	Field sera	<2	–	–	–	–	–	–	–

a HI titer using homologous avian influenza A antigen.

b corresponds to a geometric mean titer of <1:40.

HI, hemagglutination inhibition; SPF, specific pathogen free; pp-NT, pseudovirus particle neutralization test; Exp, experimental.

### Hemagglutination inhibition

The HI test using chicken erythrocytes was performed on serum samples treated with receptor-destroying enzyme (RDE) (Nordic Biolabs) as described in detail elsewhere (31). H5N2 and H7N7 viruses were used in the HI assay and were diluted to 8 HA units. The serum was serially diluted twofold, and duplicate samples were tested in two separate experiments. Button formation was scored as evidence of HI, and the serum titers are expressed as the reciprocal of the highest serum dilution that resulted in complete inhibition of hemagglutination.

### Microneutralization assay

The MN test was evaluated by CPE in cell culture (MN-CPE) and was performed using twofold serially diluted heat-inactivated serum and 100 TCID_50_ of H5N2 or H7N7 virus according to a protocol modified from the World Health Organization (WHO) manual (31). Briefly, a serum–virus mixture was incubated on MDCK cells and monitored for CPE 72–96 h after infection, and the virus-neutralizing titer was defined as the reciprocal of the serum dilution required to inhibit virus infectivity by approximately 80%. Rabbit sera were tested in duplicate in four independent experiments. For the colorimetric MN tests, CellTiter96AQuous (Promega Corporation) or WST-1 (Roche) solution (denoted Col1 and Col2, respectively) was added to each well 72 h after infection, as stipulated in the protocols provided by the manufacturers. The absorbance was measured at 492 nm for MN-Col1 and at 405 nm for MN-Col2 in a spectrophotometer (Labsystems, Multiscan EX). The neutralizing antibody titer was determined as the reciprocal of the highest serum dilution that resulted in 80% inhibition of virus infectivity and was calculated as follows: [(A_492_ serum – A_492_ virus control) / A_492_ virus control)×100] for MN-Col1 and [(A_405_ serum – A_405_ virus control) /A_405_ virus control)×100] for MN-Col2.

### Pseudovirus particle neutralization test

Performance of the pp-NT has been described previously (39). In our study, the Anhui, Hong Kong, Indonesia, Italy, Mongolia, Vietnam, and Turkey pseudoviruses were incubated with twofold serially diluted heat-inactivated serum for 1 h at 37°C in 5% CO_2_, and then approximately 5×10^3^ 293T/17 cells were added to each well. After further incubation for 48 h, BrightGlo (Promega Corporation) was added according to the manufacturer's instructions, and relative luminescence units (RLUs) were measured in a luminometer (GloMax^®^ 96 Microplate Luminometer, Promega Corporation). The inhibitory effect of the serum on pseudovirus infectivity was calculated by fitting the percentage of inhibition of RLU values [(RLU pseudovirus control – RLU serum) / (RLU pseudovirus control) ×100] (49) to a sigmoid dose–response curve with variable slope by use of GraphPad Prism 6.0 software, as previously described (34). The virus-neutralizing titer was defined as the reciprocal of the serum dilution required to inhibit pseudotype infectivity by 80%, and such titers are presented as the geometric mean. Rabbit serum samples were tested in duplicate in three or more independent experiments, and the avian serum samples were tested in duplicate.

## Results and discussion

Ever since the first reports of HPAI virus in humans appeared in the literature (50, 51), the surveillance of wild birds has become a key element in predicting and preventing outbreaks. In the European Union, it is compulsory to report the data obtained in this context, which cover not only wild birds that are sick or dead but also those that are healthy. Accordingly, there is a continuous need for safer and less labor-intensive serological methods to help evaluate serum samples collected from both wild and domestic fowl.

**Fig. 1:**
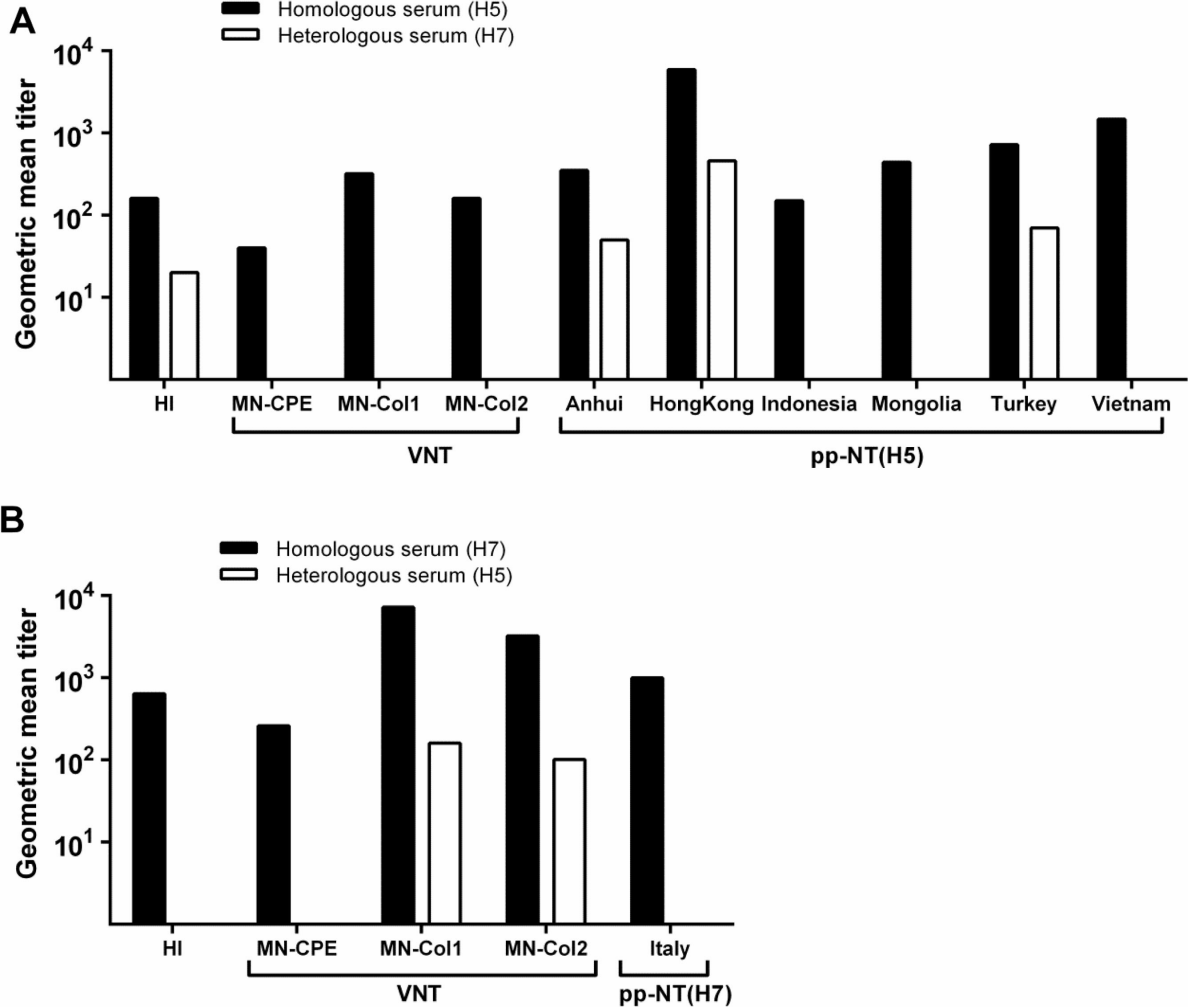
Comparison of different antibody detection assays using serum from rabbits infected with H5 and H7 influenza strains. Serological methods detecting antibodies against H5 (A) and H7 (B) viruses were evaluated using serum from rabbits infected with LPAI H5N2 or H7N7 virus. The pp-NTs were performed using six different HPAI H5 subtypes (pp-NT(H5)) and one H7 subtype (pp-NT(H7)). The results of virus neutralization tests (VNT) were determined by either CPE (MN-CPE) or colorimetric (MN-Col1 and MN-Col2) assays. Bars represent the reciprocal of the geometric mean antibody titer from two or more independent experiments that inhibited virus or pseudovirus infectivity by 80%.

Considering performance, we initially compared the different pp-NTs with standard methods such as HI and MN tests using serum from infected rabbits (Fig. 1). Each assay detecting anti-H5 or anti-H7 antibodies was evaluated using rabbit serum samples collected before infection of the animals (negative control), after infection with LPAI of subtype H5N2, or after infection with H7N7 virus (Fig. 1).

Although the HI assay does not detect specific virus-neutralizing antibodies, we included it in our study because it is one of the reference diagnostic methods recommended by the WHO for detection of IAV antibodies in animal sera (31). Homologous serum with titers of 160 and 640 inhibited hemagglutination of chicken erythrocytes by H5N2 (Fig. 1A) and H7N7 (Fig. 1B) viruses, respectively. When testing heterologous serum, cross-inhibition was observed in the HI assay based on the H5N2 virus (geometric mean titer 20, Fig. 1A) but not in HI based on H7N7 (Fig. 1B). MN testing is evaluated by visual detection of CPE in cell culture and is commonly used to detect IAV-neutralizing antibodies, and, as mentioned for the HI assay, it is recommended as a reference method by the WHO (31). In the traditional MN (MN-CPE), we found that the H5N2-positive serum had a geometric mean titer of 40, which is similar to the titers observed when using H5 MN tests based on detection of viable cells by colorimetric methods (Fig. 1A): 320 for MN-Col1 and 160 for MN-Col2. In good agreement with HI, MN-Col2 has been shown to be a reliable technique for measuring neutralizing antibodies in human serum samples before and after immunization with trivalent inactivated influenza vaccine (52). MN-Col1 has also been used to complement CPE readout and to determine antiviral activity (53). When we applied the corresponding H7 MN test (Fig. 1B), the homologous serum had a titer of 260 in MN-CPE, whereas the MN-Col1 and MN-Col2 assays were more sensitive, that is, they could detect virus-specific antibodies at considerably higher serum dilutions (titers 7240 and 3230, respectively) (Fig. 1B). Notably, cross-neutralization with heterologous serum was observed in MN-Col1 and MN-Col2 assays based on H7 (geometric mean titers 160 and 110, respectively) (Fig. 1B). Negative control sera were negative in all MN assays, except in the MN-Col2(H7) assay where the negative control sera diluted 1:80 was recorded as positive (data not shown).

Tests based on the neutralization of IAV pseudoviruses have been applied as an alternative to the MN and HI tests and have been evaluated extensively over the past few years; this has been done using serum predominantly obtained from patients (33, 34, 54, 55) but also from birds (38, 56). In our study, all six pp-NT((H5)s based on HPAI HA subtypes clearly detected the LPAI H5N2-positive rabbit serum with geometric mean titers ranging from 150 to 5890 (Fig. 1A). The Anhui, Indonesia, and Mongolia pseudoviruses had comparable titers of 350, 150, and 440, respectively, whereas the pp-NT(H5)s based on the Hong Kong, Turkey, and Vietnam pseudoviruses resulted in slightly higher geometric mean titers of 5890, 720 and 1470 (Fig. 1A). Heterologous serum was able to neutralize the Anhui and Turkey pseudoviruses at low titers (50 and 70, respectively) (Fig. 1A). Cross-neutralization was also observed in the pp-NT(H5) based on the Hong Kong pseudovirus (titer 460; Fig. 1A). All the negative control sera were negative in all ppNT(H5) and ppNT(H7) assays with the exception of Hong Kong, where control serum reacted at dilutions ranging from 1:60 to 1:480 when inhibition of 80% was set as the cutoff (data not shown). In light of the unspecific reaction in the Hong Kong pp-NT(H5), it was of interest to determine whether this feature would also be observed in serum from other species. To address that issue, we evaluated the Hong Kong pp-NT(H5) by using 23 IAV H5-negative serum samples collected from five mallards that had previously been confirmed to be H5 seronegative by ELISA and HI assessment (24). None of the serum samples were found to react in the assay at a serum dilution of 1:40 and an inhibitory concentration of 50% (data not shown), which indicates that the unspecific reactions in the assay were due to the species origin of the serum sample rather than to the assay itself.

It was recently shown that sera from chickens vaccinated with LPAI H7N1 effectively neutralized H7 pseudoviruses (38). Moreover, our pp-NT(H7) detected homologous serum at a titer of 1,000, and no cross-neutralization was observed when this test was used to analyze heterologous serum (Fig. 1B).

Our final experiments were conducted to evaluate the performances of the various pp-NTs by using a panel of avian sera (Table 1). The IAV subtype serospecificity of these serum samples had previously been confirmed by HI assay (46), and the HI titers are presented in Table 1. The pp-NT(H5)s based on Anhui, Hong Kong, Mongolia, Turkey, and Vietnam performed as well as the HI assays, recognizing all positive and negative serum samples. In some cases, these pp-NT(H5)s actually performed even better in that they detected serum at higher dilutions compared to the HI tests (Table 1). However, pp-NT(H5) based on Indonesia failed to classify one serum sample as positive; this sample had a HI titer of 64 and was collected from a turkey naturally infected with H5N2. No false positives were observed in the Hong Kong pp-NT, again pinpointing a problem with specificity when using serum from rabbits.

The pp-NT(H7) failed to recognize two serum samples, both of which were collected in the field from H7N3-infected turkeys (Table 1). These two samples tested positive with a HI titer of 16, which is close to the detection limit of the HI assay. Molesti et al. (38) noted the exact same phenomenon in several HI-positive birds that had been naturally infected with H7N3. A possible explanation for these findings is that the antibodies that were present in the investigated fowl could only inhibit hemagglutination, that is, they could not neutralize pseudotyped virus. Furthermore, it should be noted that the H7N1 and H7N7 seropositive samples obtained from experimentally infected ducks and chicken also had low pp-NT(H7) titers (100, 130, and 40) compared to the corresponding HI titers (2048, 1024, and 256), indicating that titers determined by the pp-NT(H7) are not consistently higher than HI titers.

Today, serological methods such as ELISA and the HI assay are utilized in combination with the detection of nucleic acid to maintain and confirm our knowledge of currently circulating IAV strains. Although the MN-CPE seems to be the most specific and consistent method evaluated here, the present results substantiate the promise of pp-NTs as a sensitive serological tool for global surveillance of wild birds.
